# Complete Blood Count Reference Intervals for Children Aged Less Than 1 to 12 Years in the Northern Region of Ghana

**DOI:** 10.1155/2024/6607281

**Published:** 2024-05-10

**Authors:** Gabriel Abbam, Kofi Mensah, Samuel Kwasi Appiah, Charles Nkansah, Samira Daud, Cheryl Namusoke Aikins, Akua Nyarko Osei-Afoakwa, Felix Osei-Boakye, Charles Angnataa Derigubah, Sanda Mohammed, Samuel Tandoh, Simon Bannison Bani

**Affiliations:** ^1^Department of Haematology, School of Allied Health Sciences, University for Development Studies, Tamale, Ghana; ^2^Department of Medical Laboratory Science, Faculty of Health Science and Technology, Ebonyi State University, Abakaliki, Nigeria; ^3^Department of Biomedical Laboratory Sciences, School of Allied Health Sciences, University for Development Studies, Tamale, Ghana; ^4^Department of Medical Laboratory Technology, Faculty of Applied Science and Technology, Sunyani Technical University, Sunyani, Ghana; ^5^Department of Medical Laboratory Technology, School of Applied Science and Arts, Bolgatanga Technical University, Bolgatanga, Ghana; ^6^Systems Solutions Geospatial Research Services, Accra, Ghana; ^7^Research Department, SSNIT, Accra, Ghana; ^8^University Clinic Laboratory, University of Education, Winneba, Ghana

## Abstract

Reliable laboratory diagnostic results are key for evaluating and improving children's health. To interpret these results, child-specific reference intervals (RIs), which account for constant biological changes and physiological development with sex and age, are required, as recommended by the Clinical and Laboratory Standards Institute (CLSI). This study presents age- and sex-specific reference intervals for complete blood count (CBC) parameters in children (<1–12 years old) in the Northern Region of Ghana. In this cross-sectional study, 600 healthy children from randomly sampled schools in Tamale (the Northern Region) were recruited and screened. Data from 388 eligible children were used to nonparametrically determine the reference intervals of CBC parameters at the 2.5^th^ and 97.5^th^ percentiles. The CBC reference intervals were compared for variations in sex and age groups using the Wilcoxon rank-sum test. There were no statistically significant differences in most CBC parameters by sex (RBC, Hb, HCT, MCH, RDW (CV/SD), WBC, LYM#, MON#(%) NEU#(%), EOS#(%), and BAS#(%); *p* > 0.05) and age group (RBC, MCV, RDW (CV/SD), WBC, LYM#, MON#(%) NEU#(%), EOS#(%), and BAS%; *p* > 0.05). However, there were observable differences between this locally established CBC reference interval and that used for children at Tamale Teaching Hospital (manufacturer's RIs). This study emphasises the importance of determining reference intervals representative of the local child population and incorporating them into the current reporting system of laboratories in the Northern Region to ensure the provision of effective and efficient healthcare services.

## 1. Introduction

In most clinical situations, decisions regarding patient management, diagnosis, treatment progression, clinical trials, and legal issues are made using information from routinely requested medical laboratory tests [1]. This emphasises the need for reliable and accurate results with accompanying reference intervals (RIs) that are representative of a defined population to aid in better interpretation and appropriate clinical decisions [2–5]. Haematological RIs, which provide the basis for comparison and make meaning of test results, are influenced by sex, race/genetics, age, social lifestyle/environment, and the geographical origin of the population [6]. Children undergo constant biological changes and develop physiologically at different stages in their lives. The haematopoietic system also evolves with age throughout the lifespan of an individual; thus, it is important not to treat children as “small adults” but to use representative and age-appropriate RIs when making diagnostic decisions in the clinical management of children [7–12]. Owing to the variations in RIs which result from these influences, the Clinical and Laboratory Standards Institute (CLSI) has recommended and provided guidelines for determining population-specific RIs in individual laboratories [13, 14]. Some studies in Ghana have established RIs for biochemical and haematological parameters; however, most of these studies have focused on the adult population, with only one of the studies focusing on children and adolescents in a municipality in 2014 [12]. Most medical laboratories in Ghana tend to report complete blood count (CBC) results for children with RIs from the origin of the manufacture of various brands of haematology analysers and sometimes RIs of adults without recourse to their damaging implications in clinical practice [5, 15]. As part of a series of studies aimed at establishing a national population-based haematological RI, this study presents a region-specific CBC reference interval for apparently healthy children aged less than 1-12 years in the Northern Region of Ghana, with the hypothesis that there are variations in CBC reference intervals between children of different age groups and geographic origins.

## 2. Materials and Methods

### 2.1. Ethical Approval

The Institutional Review Boards of the University for Development Studies (UDS/RB/104/23) and Tamale Teaching Hospital (TTH) (TTH/R&D/SR/135) approved the study protocol. The District Directorate of the Ghana Education Service in Tamale also approved the use of various schools as sampling centres. The objectives and advantages of the study were clarified to the parents/guardians of participants. Verbal and written consent were obtained before the children participated in the study.

### 2.2. Study Design/Area/Site

**Figure 1:**
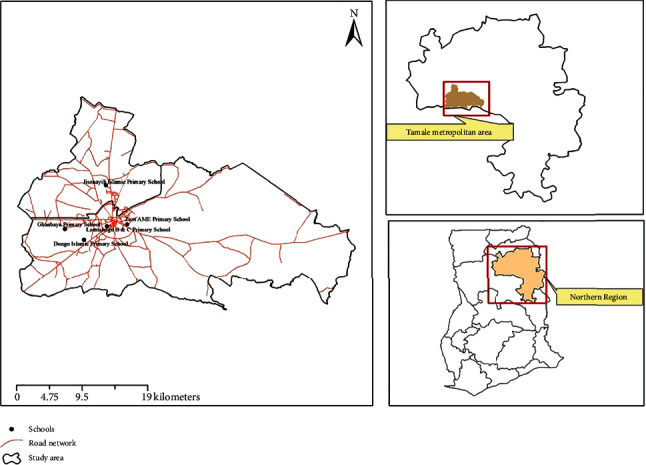
Map of Ghana created with ArcGIS showing the Tamale metropolis (study area) and the selected schools (sampling site) in the Northern Region. Source of the base map: https://data.gov.gh/dataset/shapefiles-all-districts-ghana-2012-216--260districts.

This study was conducted between February and August 2023, using a cross-sectional design to determine the CBC RIs for apparently healthy children in the Northern Region. The Tamale metropolis, which is also the capital of the Northern Region, served as the study area, with primary schools across the metropolis as sampling sites which were randomly selected to ensure comprehensive coverage. The Northern Region of Ghana is one of the most sparsely populated regions, with the Tamale metropolis situated in the central part, covering an area of approximately 646.901 square kilometres, from latitude 9°16 to 9°34 north, and spanning longitudes 0°36 to 0°57. It shares boundaries with the Sagnarigu municipality to the west and north, the Mion District to the east, the East Gonja District to the south, and the Central Gonja District to the southwest and is composed of 115 communities. The population of children in the Tamale metropolis is approximately 147,069, comprising 75,092 males and 71,973 females [16, 17]. The selected schools include Gbanbaya Primary School (with 504 pupils population) in Gbanbaya at latitude 9°23′21.45^″^N and longitude 0°54′15.73^″^W, Jisonayili Islamic School (with the population of 658 pupils) located in Kumbumgu-Jisonayili at latitude 9°26′55.80^″^N and longitude 0°51′2.82^″^W, Zion AME Primary School located in Kukuo at latitude 9°23′43.22^″^N and longitude 0°49′23.27^″^W with the population of 420 pupils, Dungu Islamic Primary School which is located in Dungu at latitude 9°22′27.96^″^N and longitude 0°52′45.83^″^W with the population of 494 pupils, and Lamashegu B and Lamashegu C Primary Schools (with the population of 455 and 390 pupils, respectively) which also are both located in Lamashegu at latitude 9°23′34.37^″^N and longitude 0°50′58.00^″^W [18].  Figure 1 shows a map of the Northern Region, depicting the Tamale metropolitan area and selected schools.

### 2.3. Enrolment of the Reference Population, Eligibility, and Sample Size

The study involved randomly selected apparently healthy native and/or resident children aged less than 1–12 years from purposively sampled schools in the Tamale metropolis. A child's participation in this study was based on the parents/guardians demonstrating willingness by the completion and signing/thumb-printing of the consent form and the willingness of the child to provide the required blood sample volume. Additionally, a child was eligible for this study upon completing and satisfying a general health status questionnaire adapted from the CLSI Guidance Document C28A2 [2]. Participants who showed evidence of acute or chronic illness, such as respiratory, cardiovascular, hepatic, or gastrointestinal conditions, had received a blood transfusion within the past 2-3 months, and had been hospitalised within the last 1–6 months or any other findings that may undermine this study's evaluation of the significance of the CBC parameters were excluded.

**Figure 2:**
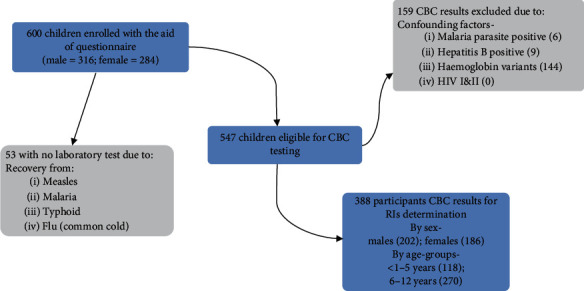
Summary of study protocol.

The CLSI guidelines for defining, establishing, and verifying RIs in clinical laboratories recommend a minimum sample size of 120 for each parameter or analyte from each sample group for the nonparametric determination of RIs [19]. Therefore, 600 healthy children from selected schools in Tamale were recruited to increase the statistical power of the study. However, the CBC results of only 388 participants were included in the determination of CBC RIs by sex, males : females (202/186), and by age groups less than 1-5 years :  6-12 years (118/270). The remaining 212 of the 600 children were excluded because of confounding factors, such as haemoglobin variants and asymptomatic malaria, as presented in Figure 2.

### 2.4. Sample Collection and Laboratory Assays

At most, 4 mL of venous blood was aseptically collected from the antecubital fossa of each child between 8:00 AM and 11:00 AM. To prevent haemolysis, the needle of the syringe was removed, 2 mL of the blood sample was dispensed into EDTA tubes (Jactermac, Germany) for haematological assays, and the remaining 2 mL was dispensed into plain glass tubes for serological assays. The sampled tubes were labelled with participant identification codes, age, and sex and transported in a cold box for analysis at the Antenatal Clinic (ANC) laboratory of the Tamale Teaching Hospital (TTH). In addition to CBC analysis, screening for confounding factors was performed to rule out conditions such as malaria, hepatitis B, sickle cells, and other abnormal haemoglobin variants.

#### 2.4.1. Complete Blood Count Analysis

White blood cell count (WBC), lymphocyte counts—absolute and percentage (LYM# and LYM%, respectively), monocyte count—absolute and percentage (MON# and MON%, respectively), neutrophil counts—absolute and percentage (NEU# and NEU%, respectively), eosinophil counts—absolute and percentage (EOS# and EOS%, respectively), basophil counts—absolute and percentage (BAS# and BAS%, respectively), red blood cell count (RBC), haemoglobin level (Hb), haematocrit (HCT), mean cell volume (MCV), mean cell haemoglobin (MCH), mean cell haemoglobin concentration (MCHC), red cell distribution widths (RDW-CV and RDW-SD), and platelet count (PLT) for all blood samples were performed within 8 hours of blood draw using URIT-5250, a 5-part haematology analyser (URIT-Medical Electronics Company Ltd., China). Daily calibration and maintenance of the haematology analyser were performed. Internal quality control (using low, normal, and high QC samples) was performed, and analyses commenced only when all quality controls were within acceptable range. The ANC laboratory of TTH also participates in the external quality assessment scheme of the United Kingdom International External Quality Assessment Scheme (UK IEQAS), in addition to its internal quality control measures.

#### 2.4.2. Confounding Factor Assessment

Sickle cell screening and haemoglobin phenotypes were investigated in each participant using 2% sodium metabisulphite and alkaline electrophoresis at pH 8.6. Rapid diagnostic test kits were used to screen for hepatitis B (Biotech Co., Ltd. Guangzhou Wondfo, China), HIV (first response HIV-I&II card test; Premier Medical Corporation Ltd., India, and oral quick by OraSure Technologies, Inc., Bethlehem, PA, USA), and malaria (Alere™ Malaria Ag P.f Ultra-sensitive RDT; 05FK140, Abbott, USA). Additionally, a 10% Giemsa-stained thick film was used to visually detect the malarial parasites under a microscope.

### 2.5. Data Management and Statistical Analysis

The study questionnaire was programmed into KoBoToolbox (https://www.kobotoolbox.org/), a web-based data collection software for ease of data collection and protection. Data were exported from the KoBoToolbox into Microsoft Excel spreadsheet and verified. Stata version 17.0 (Stata Corp, College Park, TX, USA) was used for the statistical analysis. The data were stratified by sex and age group (< 1-5years and 6-12 years). The RIs were nonparametrically determined at the 2.5^th^ and 97.5^th^ percentiles in accordance with CLSI Guidance Document C28A2, after the data of the measured CBC parameters did not conform to the Gaussian distribution when the Kolmogorov–Smirnov test for normality was performed. Outliers were estimated using the absolute difference between the most extreme distribution and the next value (*D*) and range (maximum-minimum) (*R*). Outliers were retained when *D*/*R* < 0.33 [20]. Differences in the CBC parameters by sex and age group were compared using the Wilcoxon rank-sum test. Statistical significance was set at *p* < 0.05.

## 3. Results

The 388 apparently healthy eligible participants (202 males and 186 females), who constituted the sample group for RI determination, were of the haemoglobin A phenotype and had an age range between 1 year and 12 years, with a median age of 7 years. Their basic educational status included 115 kindergartens (29.64%), 159 lower primary schools (40.98%), and 114 upper primary schools (29.38%).

### 3.1. CBC Reference Intervals by Sex

Table 1 (S1 Table) presents the nonparametrically determined 2.5^th^ and 97.5^th^ percentile CBC reference intervals, stratified by sex. RBC (*p* = 0.3282), Hb (*p* = 0.1426), HCT (*p* = 0.9909), MCH (*p* = 0.9093), RDW-CV (*p* = 0.0693), and RDW-SD (*p* = 0.266) showed no statistically significant differences between the sexes, except for MCHC, which was significantly higher in males than in females (*p* = 0.0194), and MCV and PLT which were significantly higher (*p* = 0.0170 and *p* = 0.0440, respectively) in female children than in males. Moreover, except for LYM%, which was statistically higher (*p* = 0.0296) in female children than in male children, WBC count, LYM#, MON#, MON%, NEU#, NEU%, EOS#, EOS%, BAS#, and BAS% showed no statistically significant differences between both sexes (*p* > 0.05).

### 3.2. CBC Reference Intervals by Age Group

Table 2 presents the CBC reference intervals stratified by age group (<1-5 years and 6-12 years). PLT count was significantly higher in children aged less than 1–5 years (*p* < 0.0001) than in those aged 6–12 years. Conversely, Hb (*p* = 0.0341), MCH (*p* = 0.0007), and MCHC (*p* = 0.0001) levels were significantly higher in children aged 6–12 years than in those aged less than 1–5 years. The BAS# was also significantly higher in the 6–12-year group than in the less than 1–5-year group (*p* = 0.0386).

### 3.3. Percentage Out-of-Range Values of the Established CBC RIs Compared to URIT RIs

The CBC reference interval out-of-range (OOR) values in this study were calculated. The percentage of normal Tamale children (<1-12 years) whose CBC results would have been classified as abnormal if the accompanying RIs were from URIT (Medical Electronics Company Ltd., China) is displayed in Table 3.

## 4. Discussion

Reliable laboratory diagnostic results require age- and sex-specific reference intervals to aid accurate interpretation for efficient evaluation and improvement of patient health. Thus, it is important to address the overreliance on Western-established medical laboratory reference intervals in Ghana. The lack of standardised and locally derived reference intervals has led to this practice which tends to jeopardise healthcare service consistency, particularly when dealing with haematological parameters tailored to the specific age and sex of children. To discourage this trend, this study is aimed at creating a local standard for interpreting medical laboratory findings by determining the CBC reference intervals for children aged less than 1–12 years in Tamale, Northern Ghana.

This study observed no significant differences in most CBC reference intervals when compared by sex and age group, except for Hb, MCV, MCH, MCHC, PLT, and absolute basophils, similar to studies in Guinea [21] and China [22], but contrary to a study in Macedonia [23], which showed a steady increase in CBC reference intervals with age. The inability of this study to detect and exclude children who may present with mild anaemia due to thalassaemia, evaluate their nutritional status to rule out conditions such as subclinical iron deficiency anaemia, and instead rely solely on subjective health and the absence of persistent or recent diseases may account for this difference in outcomes [21, 22, 24]. This was confirmed by the statistically significant differences in Hb, MCV, MCH, and MCHC by sex and age group observed in this study [23, 25]. In addition, sex-related differences begin mainly during puberty, with females undergoing menstruation and males exhibiting increased testosterone levels. This explains why the present study recorded significant differences in only a few CBC parameters by sex and age, as all participants were preadolescent humans [12, 23, 26].

No statistically significant variation by sex or age was recorded in the WBC count and its differential parameters in this study, except for lymphocyte percentage (LYM%) and absolute basophils (BAS#). This is in contrast to similar studies in Kintampo, Ghana, where WBC counts decreased with age [12], and a rural community in Guinea [21], where WBC, basophil, and eosinophil counts varied significantly by sex. Environmental, seasonal, and climatic factors and, to some extent, multiple aggressions (infectious diseases, injury) have been linked to variations in WBC count and its differential parameters by sex and age group in which males and particular age groups of children are exposed [22, 25, 27].

Platelet (PLT) RIs were significantly higher in females than in male children and higher in age groups less than 1–5 years than in the 6–12-year group, agreeing with the Kintampo (Ghana) [12] and Gabon [28] studies, but contradicting the findings of a Ugandan study [29], which recorded a lower PLT count in children aged 1–5 years than in 6-12 years. The variation in PLT counts in children with age and sex could be due to a combination of changes that occur in the bone marrow, megakaryopoiesis, and platelet production [30].

Notable differences were observed between the CBC reference intervals in this study and those provided by the analyser manufacturer, depicting the influence of genetic/race, environment, and geographic origin on RIs [3, 31]. The RIs of the CBC parameters were lower than those of the URIT analyser, showing significant misclassification, with %OOR ranging from 2.1% to 100% when compared to the accompanying RIs from the manufacturer. Similar disproportions have been observed in some adult RI studies in Ghana [2, 20] and other African countries [4], suggesting that the proportion of normal children whose CBC results are interpreted based on the manufacturer's RIs may be erroneously classified as having anaemia.

### 4.1. Limitations

This study was limited by the fact that no nutritional assessment, high-performance liquid chromatography (HPLC), or ferritin measurement was performed on the enrolled children to rule out thalassaemia or iron deficiency. Stool samples for occult blood testing and routine examination to rule out gastrointestinal bleeding and intestinal parasite infestation, respectively, were not conducted. Furthermore, generalised application of the results should be performed cautiously, as this study is region-specific.

## 5. Conclusion

This study locally determined the CBC reference interval specific to the age and sex of children in Tamale, which will significantly aid in improving the standard of healthcare management in Northern Ghana. Sex-, age-, and geographic-related differences were apparent, highlighting the importance of using localised population-specific reference intervals in clinical practice. Similar CBC RI studies are recommended for children in other regions of the country.

## Figures and Tables

**Table 1:** CBC reference intervals of Tamale children by sex.

Parameters	Unit	Combined RIs of children			Males			Females			*p* value
		*N*	Median	Reference values	*N*	Median	Reference values	*N*	Median	Reference values	
RBC	10^6^/*μ*L	379	4.28	3.58-5.04	196	4.33	3.58-5.09	183	4.26	3.57-5.03	0.3282
Hb	g/dL	376	11.10	9.40-12.56	195	11.20	9.80-12.60	181	11.10	9.12-12.40	0.1426
HCT	%	377	35.30	29.50-41.41	195	35.30	29.28-41.50	182	35.4	30.16-41.64	0.9909
MCV	*μ*m^3^	379	82.5	70.45-92.85	194	81.85	68.85-91.35	185	83.3	73.01-93.87	0.0170
MCH	pg	374	25.3	20.60-29.00	190	25.4	20.19-29.10	184	25.3	20.73-29.0	0.9093
MCHC	g/dL	368	30	28.00-34.00	190	31	28.00-34.00	178	30	28.0-33.81	0.0194
RDW-CV	%	368	8.4	7.30-10.00	189	8.5	7.40-10.00	179	8.3	7.05-10.15	0.0693
RDW-SD	*μ*m^3^	380	47.5	38.71-52.68	197	46.7	39.38-52.30	183	47.5	37.96-53.90	0.266
PLT	10^3^/*μ*L	385	318	121-580	199	308	124-598	186	327	108-572	0.0440
WBC	10^3^/*μ*L	373	5.98	3.70-8.61	193	6	3.80-8.62	180	5.83	3.51-8.65	0.2943
LYM#	10^3^/*μ*L	379	2.42	1.13-3.93	195	2.38	1.36-3.91	184	2.46	0.94-3.96	0.9596
LYM%	%	381	42.33	24.97-59.20	200	41.1	26.23-57.40	181	44.21	23.41-62.36	0.0296
MON#	10^3^/*μ*L	372	0.43	0.19-0.74	191	0.44	0.20-0.74	181	0.42	0.16-0.72	0.3214
MON%	%	366	7.1	3.60-10.99	190	7.07	3.73-10.83	176	7.27	3.50-11.21	0.8093
NEU#	10^3^/*μ*L	370	2.65	1.28-4.59	189	2.74	1.25-4.24	181	2.47	1.31-4.75	0.0965
NEU%	%	384	45.74	27.46-66.08	201	47.16	30.16-65.66	183	44.79	25.70-66.76	0.2687
EOS#	10^3^/*μ*L	353	0.14	0.02-0.44	177	0.13	0.03-0.45	176	0.15	0.01-0.45	0.3542
EOS%	%	345	2.25	0.43-6.70	174	2.1	0.51-6.85	171	2.43	0.36-6.65	0.5867
BAS#	10^3^/*μ*L	344	0.01	0.00-0.03	184	0.01	0.00-0.03	160	0.01	0.00-0.03	0.8366
BAS%	%	338	0.16	0.06-0.40	184	0.16	0.06-0.40	154	0.16	0.06-0.40	0.4020

RBC: red blood cells; Hb: haemoglobin; HCT: haematocrit; MCV: mean cell volume; MCH: mean cell haemoglobin; MCHC: mean cell haemoglobin concentration; RDW-CV: red cell distribution width-coefficient of variation; RDW-SD: red cell distribution width-standard deviation; PLT: platelet count; WBC: white blood cells; LYM: lymphocyte; MON: monocyte; NEU: neutrophil; EOS: eosinophil; BAS: basophil; #: absolute.

**Table 2:** CBC reference intervals of Tamale children by age group.

Parameters	Unit	<1-5 years				6-12 years				*p* value
		*N*	Median	Reference values	90% CI	*N*	Median	Reference values	90% CI^†^	
RBC	10^6^/*μ*L	112	4.32	3.60-4.91	3.54-3.76; 4.83-5.21	267	4.27	3.57-5.11	3.41-3.64; 4.95-5.20	0.5227
Hb	g/dL	114	11.00	8.89-12.40	8.80-9.40; 12.20-12.60	262	11.20	9.70-12.60	9.30-9.80; 12.40-12.60	0.0341
HCT	%	115	35.7	30.18-40.67	29.20-31.32; 40.39-41.70	262	35.20	29.36-41.67	28.55-30.10; 40.46-42.25	0.0715
MCV	*μ*m^3^	117	83.1	69.39-95.02	67.30-73.55; 90.43-96.3	262	81.85	70.49-92.83	68.70-72.20; 91.75-93.85	0.4480
MCH	pg	113	24.6	20.23-29.00	19.2-21.09; 28.11-29.0	261	25.6	20.60-29.10	19.80-21.34; 28.7-29.90	0.0007
MCHC	g/dL	102	30	28.00-33.30	27.5-28.74; 32.04-34.1	266	31	28.00-34.00	27.17-28.0; 34.00-34.10	0.0001
RDW-CV	%	112	8.3	7.28-10.25	7.0-7.4; 9.6-10.5	256	8.4	7.30-10.00	7.0-7.40; 9.80-10.26	0.9592
RDW-SD	*μ*m^3^	112	46.7	37.60-54.04	37.6-38.76; 50.83-54.7	268	47.5	39.26-52.52	38.20-40.27; 52.3-53.90	0.3394
PLT	10^3^/*μ*L	118	356	117 – 605	101-195; 574-626	267	300	123-565	104-148; 493-584	<0.0001
WBC	10^3^/*μ*L	109	6	3.88-8.60	3.4-4.14; 8.04-8.7	264	5.94	3.66-8.74	3.21-3.85; 8.4-9.04	0.9701
LYM#	10^3^/*μ*L	114	2.38	0.85-3.99	0.55-1.15; 3.91-4.27	265	2.45	1.22-3.86	1.08-1.39; 3.70-4.12	0.6171
LYM%	%	114	42.34	21.25-63.80	19.02-24.87; 58.80-66.29	267	42.26	26.44-57.93	25.09-27.19; 57.20-60.24	0.2493
MON#	10^3^/*μ*L	115	0.44	0.18-0.74	0.09-0.21; 0.69-0.8	257	0.43	0.19-0.73	0.14-0.22; 0.68-0.78	0.5967
MON%	%	111	7.07	3.26-10.89	2.9-3.58; 10.41-11.67	255	7.1	3.77-11.05	3.36-4.27; 10.81-11.41	0.4302
NEU#	10^3^/*μ*L	110	2.65	1.20-4.74	0.41-1.52; 4.25-4.87	260	2.64	1.29-4.48	1.10-1.36; 4.19-4.75	0.7046
NEU%	%	114	47.61	26.60-67.90	19.44-29.43; 64.01-69.68	270	45.09	29.04-64.91	24.34-31.23; 63.05-69.12	0.2714
EOS#	10^3^/*μ*L	111	0.13	0.01-0.44	0.01-0.02; 0.40-0.45	242	0.14	0.02-0.46	0.01-0.04; 0.41-0.47	0.5453
EOS%	%	108	2.11	0.18-6.77	0.13-0.53; 5.88-7.15	237	2.32	0.56-6.70	0.23-0.68; 6.53-7.26	0.3273
BAS#	10^3^/*μ*L	100	0.01	0.00-0.03	0-0; 0.03-0.03	244	0.01	0.00-0.03	0.00-0.00; 0.02-0.03	0.0386
BAS%	%	98	0.16	0.06-0.42	0.04-0.06; 0.36-0.42	240	0.16	0.06-0.38	0.06-0.06; 0.35-0.4	0.8249

RBC: red blood cells; Hb: haemoglobin; HCT: haematocrit; MCV: mean cell volume; MCH: mean cell haemoglobin; MCHC: mean cell haemoglobin concentration; RDW-CV: red cell distribution width-coefficient of variation; RDW-SD: red cell distribution width-standard deviation; PLT: platelet count; WBC: white blood cells; LYM: lymphocyte; MON: monocyte; NEU: neutrophil; EOS: eosinophil; BAS: basophil; #: absolute; CI^†^: confidence interval values for lower and upper limits.

**Table 3:** CBC out-of-range (OOR) values based on comparison with URIT (Medical Electronics Company Ltd., China) values.

Parameter	Unit	URIT-5250 child RIs	%OOR
RBC	10^6^/*μ*L	3.50-5.20	4.1
Hb	g/dL	12.0-16.0	82.2
HCT	%	35.0-49.0	46.4
MCV	*μ*m^3^	80.0-100.0	37.6
MCH	pg	27.0-34.0	80.7
MCHC	g/dL	31.0-37.0	54.6
RDW-CV	%	11.0-16.0	100
RDW-SD	*μ*m^3^	35.0-56.0	2.1
PLT	10^3^/*μ*L	150-450	18.5
WBC	10^3^/*μ*L	4.0-12.0	8.2
LYM#	10^3^/*μ*L	0.8-7.0	2.8
LYM%	%	20.0-60.0	3.6
MON#	10^3^/*μ*L	0.12-1.2	4.4
MON%	%	3.00-10.00	15.5
NEU#	10^3^/*μ*L	2.0-8.0	25.3
NEU%	%	50.0-70.0	66.2
EOS#	10^3^/*μ*L	0.02-0.80	10.8
EOS%	%	0.50-5.00	27.8
BAS#	10^3^/*μ*L	0.00-0.10	11.3
BAS%	%	0.00-1.00	12.9

## Data Availability

All relevant data are available in this article and its supplementary file.

## Supplementary Materials

Supplementary Materials S1 Table: CBC reference intervals of Tamale children by sex.
