# Supplementary material for: Complete Blood Count Reference Intervals for Children Aged Less Than 1 to 12 Years in the Northern Region of Ghana
Source: Biomed Res Int. 2024 May 10;2024:6607281. doi: 10.1155/2024/6607281 (PMC11101252; doi:10.1155/2024/6607281)
Supplement: Supplementary Materials — S1 Table: CBC reference intervals of Tamale children by sex. [file 6607281.f1.pdf]

S1 Table. CBC reference intervals Tamale children by sex

Parameters	Combined			
	**N**	**Median**	**Reference Values**	**90% C.I†**
RBC (10^6/µL)	379	4.28	3.58 - 5.04	3.49-3.65; 4.93-5.16
Hb (g/dL)	376	11.1	9.40 - 12.56	9.05-9.70; 12.40-12.60
HCT (%)	377	35.3	29.50 - 41.41	29.14-30.13; 40.53-42.06
MCV (µm^3)	379	82.5	70.45 - 92.85	68.67-71.92; 91.58-93.96
MCH (pg)	374	25.3	20.60 - 29.00	19.83-21.1; 28.7-29.26
MCHC (g/dL)	368	30	28.00 - 34.00	27.63-28; 34-34
RDW-CV (%)	368	8.4	7.30 - 10.00	7.02-7.4; 9.80-10.27
RDW-SD (µm^3)	380	47.5	38.71 - 52.68	37.6-39.54; 52.3-53.9
PLT (10^3/µL)	385	318	121-580	105-144; 538-598
WBC(10^3/µL)	373	5.98	3.70 - 8.61	3.4-3.9; 8.38-8.9
LYM # (10^3/µL)	379	2.42	1.13 - 3.93	0.92-1.23; 3.83-4.06
LYM %	381	42.33	24.97 - 59.20	21.46-26.54; 57.59-62.56
MON # (10^3/µL)	372	0.43	0.19 - 0.74	0.14-0.21; 0.69-0.78
MON %	366	7.1	3.60 - 10.99	3.22-3.78; 10.77-11.29
NEU # (10^3/µL)	370	2.65	1.28 - 4.59	1.12-1.37; 4.28-4.76
NEU %	384	45.74	27.46 - 66.08	24.34-29.96; 66.55-68.74
EOS # (10^3/µL)	353	0.14	0.02 - 0.44	0.01-0.03; 0.41-0.46
EOS %	345	2.25	0.43 - 6.70	0.22-0.61; 6.54-7.15
BAS # (10^3/µL)	344	0.01	0.00 - 0.03	0-0; 0.03-0.03
BAS %	338	0.16	0.06 - 0.40	0.06-0.06; 0.37-0.42
	**Males**			
RBC (10^6/µL)	196	4.33	3.58 - 5.09	3.39-3.69; 4.90-5.19
Hb (g/dL)	195	11.2	9.80 - 12.60	9.70-9.90; 12.49-12.60
HCT (%)	195	35.3	29.28 - 41.50	28.25-30.01; 40.59-41.95
MCV (µm^3)	194	81.85	68.85 - 91.35	67.3-71.10; 89.5-93.66
MCH (pg)	190	25.4	20.19 - 29.10	19.64-21.07; 28.61-30.11
MCHC (g/dL)	190	31	28.00 - 34.00	27.42-29; 34-34.57
RDW-CV (%)	189	8.5	7.40 - 10.00	7.3-7.5; 9.8-10.26
RDW-SD (µm^3)	197	46.7	39.38 - 52.30	38.13-41.01; 51.552.68
PLT (10^3/µL)	199	308	124 - 598	102-146; 535-618
WBC(10^3/µL)	193	6	3.80 - 8.62	3.23-4.03; 8.30-9.11
LYM # (10^3/µL)	195	2.38	1.36 - 3.91	1.13-1.51; 3.71-4.30
LYM %	200	41.1	26.23 - 57.40	22.22-27.15; 55.78-58.29
MON # (10^3/µL)	191	0.44	0.20 - 0.74	0.14-0.24; 0.70-0.78
MON %	190	7.07	3.73 - 10.83	2.84-4.26; 10.55-11.28
NEU # (10^3/µL)	189	2.74	1.25 - 4.24	1.02-1.40; 3.99-4.65
NEU %	201	47.16	30.16 - 65.66	23.32-31.47; 62.82-69.39
EOS # (10^3/µL)	177	0.13	0.03 - 0.45	0.01-0.05; 0.41-0.47
EOS %	174	2.1	0.51 - 6.85	0.15-0.65; 6.58-7.25
BAS # (10^3/µL)	184	0.01	0.00 - 0.03	0-0; 0.02-0.03

BAS %	184	0.16	0.06 - 0.40	0.05-0.06; 0.38-0.42
**Females**				
RBC (10^6/µL)	183	4.26	3.57 - 5.03	3.48-3.67; 4.91-5.25
Hb (g/dL)	181	11.1	9.12 - 12.40	8.80-9.40; 12.20-12.60
HCT (%)	182	35.4	30.16 - 41.64	29.26-31.13; 40.23-42.30
MCV (µm^3)	185	83.3	73.01 - 93.87	70.38-74.37; 92.05-85.97
MCH (pg)	184	25.3	20.73 - 29.0	20.04-21.47; 28.63-29.26
MCHC (g/dL)	178	30	28.0 - 33.81	27.08-28; 33.05-34.17
RDW-CV (%)	179	8.3	7.05 - 10.15	7-7.4; 9.74-10.5
RDW-SD (µm^3)	183	47.5	37.96 - 53.90	37.6-39.24; 52.3-54.7
PLT (10^3/µL)	186	327	108 - 572	102-168; 521-582
WBC(10^3/µL)	180	5.83	3.51 - 8.65	3.05-3.9; 8.20-9.04
LYM # (10^3/µL)	184	2.46	0.94 - 3.96	0.58-1.20; 3.82-4.22
LYM %	181	44.21	23.41 - 62.36	19.73-26.32; 59.17-66.47
MON # (10^3/µL)	181	0.42	0.16 - 0.72	0.10-0.21; 0.64-0.8
MON %	176	7.27	3.50 - 11.21	2.98-3.69; 10.79-11.67
NEU # (10^3/µL)	181	2.47	1.31 - 4.75	0.63-1.41; 4.48-4.88
NEU %	183	44.79	25.70 - 66.76	19.81-29.53; 64.54-70.41
EOS # (10^3/µL)	176	0.15	0.01 - 0.45	0.01-0.02; 0.37-0.48
EOS %	171	2.43	0.36 - 6.65	0.14-0.65; 5.84-7.35
BAS # (10^3/µL)	160	0.01	0.00 - 0.03	0-0; 0.03-0.03
BAS %	154	0.16	0.06 - 0.40	0.06-0.07; 0.32-0.42

CI†: confidence interval values for lower and upper limits.
